# Rapid diagnosis of herpes simplex virus 1 and 2 bloodstream infections utilizing a sample-to-answer platform

**DOI:** 10.1128/jcm.00131-24

**Published:** 2024-08-12

**Authors:** Wei Zhen, Farah Sheikh, Dwayne A. Breining, Gregory J. Berry

**Affiliations:** 1 Infectious Disease Diagnostics, Northwell Health Laboratories, Lake Success, New York, USA; 2 Department of Pathology and Laboratory Medicine, Donald and Barbara Zucker School of Medicine at Hofstra/Northwell, New York, New York, USA; The University of North Carolina at Chapel Hill School of Medicine, Chapel Hill, North Carolina, USA

**Keywords:** bloodstream herpes simplex virus infections, disseminated HSV, rapid diagnostic testing, sample-to-answer platform, rapid turnaround time

## Abstract

Bloodstream HSV-1 and HSV-2 infections can cause devastating outcomes with high morbidity and mortality, especially in neonates or immunocompromised individuals. Proper patient management for herpes simplex virus (HSV) bloodstream infections is time-sensitive and requires a rapid, accurate, and definitive diagnosis. The absence of the U.S. Food and Drug Administration (FDA)-approved molecular assays for HSV detection in blood, coupled with a lack of consensus on the optimal sample type, underscores the unmet need for improved diagnostics. We prospectively compared the cycle threshold values in paired samples including whole blood (WB), plasma, serum, and peripheral blood mononuclear cells (PBMCs) from patients with bloodstream HSV infections. This analysis employed a modified use of the FDA-cleared Simplexa HSV-1 & 2 Direct assay. The clinical performance in serum was assessed by comparing the results of 247 remnant specimens on this sample-to-answer platform to established laboratory-developed tests in a blinded fashion. Serum samples exhibited significantly lower cycle thresholds than whole blood samples [2.6 cycle threshold (Ct) bias, *P* < 0.001]. The modified Simplexa assay demonstrated 100% positive percent agreement for the detection of HSV-1 and HSV-2 DNA in serum samples and yielded an overall agreement of 95% (95% CI, 0.92 to 0.97), with a κ statistic of 0.75 (95% CI, 0.62 to 0.86) compared to the composite reference method. Discordance rates were 5.20% for HSV-1 and 0.81% for HSV-2. This investigation demonstrates that serum is an optimal specimen type for HSV detection when compared to several blood compartments. Serum offers a promising sample type for rapid and accurate diagnosis of HSV bloodstream infections using the modified Simplexa assay.

**IMPORTANCE:**

Rapid, accurate, and definitive diagnosis of herpes simplex virus (HSV) infections is crucial in clinical settings for patient management. The absence of FDA-authorized molecular assays for HSV-1/2 detection in blood, coupled with a lack of consensus on the optimal sample type, underscores the need for improved diagnostic methods. Furthermore, rapid diagnosis of HSV bloodstream infections enables timely administration of antiviral treatment, influences patient management decisions for those at high risk, and can contribute to shorter hospital stays, thereby reducing healthcare costs.

## INTRODUCTION

Herpes simplex viruses are among the most ubiquitous in human infections. In most cases, HSV infections in individuals with an intact immune system are relatively mild and can cause discomfort, but often go unnoticed. In rare instances, HSV can enter the bloodstream, leading to more severe manifestations affecting multiple regions of the skin, the viscera, and the central nervous system. This is especially true for neonates and individuals with an immunocompromised status, for whom HSV infections or reactivations can be life-threatening (1–6). Neonatal disseminated HSV infection, for example, can result in devastating outcomes, with an untreated mortality rate of approximately 80% and up to 30% even with antiviral therapy (7–10). In solid organ transplantation recipients, HSV infection or reactivation can also lead to more severe manifestations, including esophagitis, hepatitis, pneumonitis, and potential graft loss (11–14). Therefore, achieving a rapid, accurate, and definitive diagnosis of HSV bloodstream infections is crucial in clinical settings (5, 6, 15, 16). Rapid diagnosis enables timely administration of antiviral treatment, influences patient management decisions for those at high risk, and can contribute to shorter hospital stays, thereby reducing healthcare costs (17, 18).

Various methods have been employed for the diagnosis of HSV-1 and HSV-2 infections including conventional viral culture, serological tests, and nucleic acid amplification tests (NAAT) (19–21). Among these diagnostic methods, polymerase chain reaction (PCR) is widely utilized due to its high sensitivity and specificity, as well as its short turnaround time for detecting viral nucleic acid (11, 15, 16, 19). However, it is important to note that FDA-cleared assays for HSV-1 and HSV-2 detection are currently limited to two specimen types, cerebrospinal fluid (CSF) and cutaneous or mucocutaneous lesion swabs (19, 21).

The American Academy of Pediatrics recommends that all infants born to mothers who test positive for genital HSV lesions undergo testing for HSV within 24–36 h of birth. This includes PCR testing in whole blood and HSV cultures (22). Presently, no FDA-cleared molecular assays are specifically designed for HSV-1 and HSV-2 detection in blood as a specimen type. However, HSV NAAT laboratory-developed tests (LDTs) using blood are commonly used in clinical practice for the management of patients suspected of HSV-1 and HSV-2 infections. Previous studies have explored various sample types for HSV detection, including whole blood, dried blood spots, serum, plasma, and peripheral blood mononuclear cells (PBMC) (16, 23–29). However, there is a lack of standardization and consensus on the optimal specimen type for detecting bloodstream HSV infections. While whole blood is widely utilized, there is currently insufficient data to demonstrate its superiority over other blood compartments for diagnosis and managing HSV-1 and HSV-2 infections. Therefore, the aim of this study was to investigate whether there were significant differences in HSV-1 and HSV-2 DNA detection in whole blood, plasma, serum, and PBMC-paired samples collected from patients with HSV infection to determine the ideal clinical sample type to use for HSV detection in blood. We used the DiaSorin Simplexa HSV-1 & 2 Direct assay system for this analysis since it is a simple and rapid sample-to-answer platform that could fulfill the need for rapid diagnosis and monitoring in HSV-positive patients (30). Additionally, we evaluated the analytical and clinical performance of HSV-1 and HSV-2 DNA detection and differentiation in the bloodstream by comparing the results of the modified Simplexa HSV-1 and 2 Direct assay to those of a composite reference method.

## MATERIALS AND METHODS

### Study design

A total of 18 samples were gathered from patients ranging in age from 23 to 93 years, all of whom were initially HSV-positive in samples obtained from lesions, cerebrospinal fluid (CSF), or whole blood (WB). During the collection of qualified samples from HSV-positive lesions or CSF, we reviewed each patient’s Electronic Medical Record (EMR) within the Laboratory Information System (LIS) to identify if an additional EDTA whole blood sample and/or serum (collected in a gold-top tube) had been ordered and collected simultaneously from the same patient. Upon identifying such cases, the serum or whole blood samples were tested for the presence of HSV-1 or HSV-2 DNA using the modified Simplexa HSV-1 & 2 Direct assay (modified Simplexa assay), which has been previously validated at Northwell Health Laboratories for whole blood specimens (16). In cases where either serum or whole blood samples tested positive for HSV, all other blood compartments from the same patient, including serum, plasma, WB, and PBMC, were prepared. For patients who initially tested positive for HSV in whole blood, the EMR/LIS was used to locate available remnant serum samples or to procure another whole blood sample if the initial quantity was insufficient to confirm that both serum and WB samples were collected simultaneously for additional routine laboratory testing. This comprehensive analysis encompassed various blood compartments, with HSV results and cycle threshold (Ct) values generated using the modified Simplexa assay (16).

To evaluate the performance of PCR using different sample types (whole blood, plasma, serum, and PBMC), a minimum of 2.5 mL of whole blood specimen was required from each patient. The preparation of blood fractions involved the following steps: (i) A 300 µL aliquot of whole blood was diluted 1:1 with 300 µL phosphate-buffered saline (PBS) without Mg^2+^ and Ca^2+^ (PBS) for the modified Simplexa assay; (ii) the remaining blood was used for the isolation of plasma and PBMC. The BD vacutainer CPT cell preparation tube with Sodium citrate (Reference number: 362761, Becton Dickinson, Franklin Lakes, NJ) was employed for PBMC isolation. Briefly, the top fluid layer containing the anticoagulant was removed using a sterile transfer pipette. Next, 2.0 mL of PBS without Mg^2+^ and Ca^2+^ was added to rinse away the residual anticoagulant. Following the removal of the PBS, a minimum of 2.0 mL of EDTA whole blood was added to the CPT tube. After centrifugation at 1,800 RCF for 30 min at room temperature, the plasma fraction above the PBMC layer was collected and transferred to the second tube for testing. Additional diluted plasma was prepared by mixing the plasma with PBS in a 1:1 dilution. The cell layer containing PBMC was rinsed with 5.0 mL RPMI-1640 and transferred to a 15.0 mL conical tube; following centrifugation, the cell pellet was rinsed twice by adding 12.0 mL of PBS. The final rinse was centrifuged at 300 RCF for 5 min at room temperature. The supernatant was then removed, and PBS was added to achieve a final volume of 1.0 mL. A cell concentration and cell viability count were performed using the Muse Cell Analyzer (Muse Count & Viability Kit MCH100102; EMD Millipore, Billerica, MA, USA). Samples where cell viability was greater than 85%, and the isolated PBMC had a concentration of at least 2.0 × 10^6^ cells/mL were analyzed.

A total of 247 residual serum specimens were prospectively collected between June 2019 and June 2023 from patients with a confirmed HSV-1 or HSV-2 lesion-positive infection, as determined by the Lyra HSV-1/2 and VZV assay (Quidel Corp, San Diego, CA). These specimens were collected from 23 hospitals affiliated with the Northwell Health System. Following de-identification for each collected specimen, the same specimen was divided into two aliquots and stored in an ultra-low freezer (−80°C ± 10°C); One set of aliquots underwent testing using the modified Simplexa assay, while the other set was sent to DiaSorin Molecular where a comparator assay was performed in a blinded fashion.

### Detection and differentiation of HSV-1 and HSV-2 using modified Simplexa HSV-1 & 2 Direct assay

In this study, detection and differentiation of HSV DNA was conducted using a modification of the FDA-cleared DiaSorin Molecular Simplexa HSV-1 & 2 Direct assay system, which consists of the Simplexa HSV-1 & 2 Direct assay kit, the LIAISON MDX instrument, the 8-well Direct Amplification Disc (DAD) and associated accessories (DiaSorin Molecular LLC, Cypress, CA, USA). The assay loading, cycling parameters, and data analyses are identical to steps with the modified Simplexa assay that have previously been validated (16). An internal control included in the Simplexa HSV-1 & 2 Direct Kit was used to monitor PCR failure and/or inhibition. The assay process involved adding 50 µL of serum or plasma directly without further preparation or extraction, diluted whole blood, diluted plasma, or PBMC to the “SAMPLE” well of the DAD followed by the addition of 50 µL of Simplexa HSV-1 & 2 Direct Kit reaction mix to the “R” well of the DAD. Assays were run on the LIAISON MDX platform, utilizing the following cycling parameters: an initial denaturation at 97°C for 120 s, followed by 42 amplification cycles (10 s at 97°C and 30 s at 60°C). For the HSV-1 target, samples with a Ct value <40 were deemed positive; for the HSV-2 target, samples with a Ct value <42 were considered positive. Data collection and analysis were performed with LIAISON MDX studio software. For quality control purposes, in-house HSV-1 and HSV-2 combined positive control and negative control were included in all testing procedures.

### Limit of detection

The limit of detection (LOD) of the assay in serum was determined by testing serial dilutions of quantified and inactivated HSV-1 (MacIntyre strain, catalog number: NATHSV1-0005; ZeptoMetrix, Buffalo, NY) and HSV-2 (MS strain, catalog number: NATHSV2-0005; ZeptoMetrix, Buffalo, NY). HSV-1 and HSV-2 viral particles were provided at a concentration of 59,100 copies/mL and 50,900 copies/mL, respectively. Serial dilutions were prepared using a pooled human serum that confirmed negative for HSV-1 and HSV-2 DNA (material number: 1830-0002; Seracare, Milford, MA) to achieve the following concentrations in copies/mL: 20,000, 400, 200, 100, and 50. Multiple replicates ranging from 5 to 12 at each dilution were tested using the modified Simplexa assay. The LOD was defined as the concentration of the lowest dilution that could be detected with a probability greater than 95% and was determined by probit analysis.

### Precision (intra and inter-assay reproducibility)

The precision of the assay for HSV detection in serum was assessed using HSV-1 and HSV-2 whole virus diluted in pooled human negative serum. Both inter-assay and intra-assay reproducibility were evaluated. Three spiked serum samples containing HSV-1 and HSV-2 were utilized, sourced from the LOD study. These samples consisted of two concentrations at or near the LOD and one with a relatively high titer. Inter-assay reproducibility was assessed by testing these samples on three different days by two different technologists, while intra-assay reproducibility was evaluated by testing three spiked HSV-1 and HSV-2 samples in triplicate. The study employed two LIAISON MDX instruments and two lots of assay reagents.

### First comparator method—PCR and bi-directional sequencing (LDT 1)

The performance of the modified Simplexa assay was compared to a PCR and bi-directional sequencing laboratory-developed test (LDT 1). This was composed of a validated real-time PCR assay for HSV-1 and HSV-2, followed by confirmation of positive PCR amplification products through bi-directional sequencing. The real-time PCR and bi-directional sequencing assays were specifically designed to amplify well-conserved regions of the HSV-1 and HSV-2 DNA polymerase genes (31) and to identify HSV-1 and HSV-2 DNA using SYBR Green PCR chemistry. SYBR green melt curve data were utilized to confirm the presence of the HSV amplicon. This analysis process involved four steps. (i) DNA extraction from 200 µL of sample and eluted in 50 µL using a Magna Pure 96 instrument (Roche Diagnostics Corporation, Indianapolis, IN), with the addition of 5 µL of internal control (32), which contains non-HSV-1 and HSV-2 related sequences; (ii) 10 µL of extracted sample was employed for PCR amplification, utilizing SYBR Green PCR (QIAGEN, Redwood City, CA) on an ABI 7500 real-time PCR instrument (Thermo Fisher Scientific, Waltham, MA). Positive samples were identified by the generation of an amplicon with a defined melting temperature; (iii) sequencing of PCR amplicons in both directions using the BigDye cycle sequencing chemistry (Life Technologies, Carlsbad, CA, USA); (iv) Comparison of bi-directional sequencing results against the GenBank database via BLAST search to determine whether the sample tested positive for HSV-1 or HSV-2. Additionally, a separate PCR for an internal control was set up to monitor extraction and PCR inhibition and to validate negative HSV results. The LOD of LDT 1 was previously determined to be 1,000 copies/mL for HSV-1 and 500 copies/mL for HSV-2.

### Second comparator method for discordant resolution (LDT 2)

Discordant results between LDT 1 and the modified Simplexa assay were further analyzed using a qualitative real-time PCR method which was developed and validated by DiaSorin Molecular and designated as a laboratory-developed test (LDT 2). LDT 2 was specifically designed to amplify and detect a discrete region of the HSV-1 and HSV-2 DNA polymerase. Briefly, 5 µL of internal control (32) was added to 200 µL of each specimen followed by extraction using the MagNa Pure 96 instrument (Roche). For each amplification reaction, 5 µL of reaction mix (composed of 4 µL of Universal Master Mix, 0.2 µL HSV-1 Primer Pair, 0.3 µL HSV-2 Primer Pair, 0.3 µL DI water and 0.2 µL Simplexa extraction, and amplification control primer pair) was combined with 5 µL extracted specimen in the 96-well Universal Disc. Assays were run on the LIAISON MDX with the following cycling parameters: 120 s at 97°C initial denaturation followed by 40 cycles of amplification (10 s at 97°C and 30 s at 60°C). A sample with a Ct value <40 was considered positive. The limit of detection of this method was previously determined to be 250 copies/mL for HSV-1 and 500 copies/mL for HSV-2.

### Composite reference method

The composite reference method (CRM) used in this study was a combination of results from LDT 1 and LDT 2 in which one of the two assays was positive. This was used as a “gold standard” to compare to the modified Simplexa assay result.

### Statistical analysis

The analysis of positive percent agreement (PPA), negative percent agreement (NPA), Probit, paired analysis, and two-sided (upper/lower) 95% confidence interval (CI) was performed using Microsoft Office Excel 365 MSO software (Microsoft, Redmond, WA) and Prism GraphPad software (GraphPad Software Inc., San Diego, CA, USA). The percent positive agreement was calculated as TP/(TP + FN) × 100, and the percent negative agreement was calculated as TN/(TN + FP) × 100, where TP was true-positive, FN was false-negative, TN was true-negative, and FP were false-positive results. Probit analyses were used for the copies/mL determination of the analytical sensitivity study. The dose-response 95th percentile (with 95% CI) model was assessed using the Finney and Stevens calculations (33). The coefficient of variation (as a percentage, CV%) was determined by dividing the standard deviation of the mean divided by the mean value multiplied by 100. Cohen’s kappa values (κ) were calculated as a measure of the overall agreement, categorized as almost perfect (>0.90), strong (0.80 to 0.90), moderate (0.60 to 0.79), weak (0.40 to 0.59), minimal (0.21 to 0.39), or none (0 to 0.20). The discordance rate was calculated as (FP + FN)/total number of samples tested × 100.

## RESULTS

### Comparison of HSV detection and cycle threshold values in paired blood samples

A total of 18 samples from patients with known PCR-confirmed HSV-positive results were tested for the presence of HSV-1 and HSV-2 DNA in blood or serum using the Simplexa Direct assay, and Ct values were compared in a subset of these 18 samples where paired WB, plasma, serum, or PBMC samples were available (Table 1). As shown in Table 1, HSV-1 or HSV-2 DNA was detected across different blood compartments, ranging from 18.8% (3/16 in PBMC) to 100% (14/14 in serum) of patients. Notably, both HSV-1 and HSV-2 DNA were detected in the serum of all six patients presenting HSV-positive lesions, with a detection rate of 100%. The detection rates for HSV DNA were comparable in whole blood and diluted PBS plasma, with a detection rate of 94.4%. Furthermore, the two HSV-1 samples, for which HSV-1 was not detectable in whole blood or plasma, exhibited positive detection in serum, each with a Ct value of 38.6. Poor detection was observed in undiluted plasma and PBMC, with a detection rate of 66.7% and 18.8%, respectively. Of note, among the undiluted plasma samples (4 out of 18), invalid results and internal control failures were observed, with internal control amplification occurring in only 77.8% (14 out of 18) of the samples tested. Four samples with initially invalid results were also invalid upon repeat, likely due to existing interfering instances in plasma, and this observation aligns with the findings from an article by Kuypers (34), which reported a high invalid rate with plasma samples using the Simplexa HSV-1 & 2 Direct assay.

**TABLE 1:** HSV-1 and 2 detection and differentiation across several blood compartments in patients with HSV infection, with Ct values generated from testing via the modified Simplexa assay

Sample ID	Analyte^*a*^	Initial positive specimen	Age	Gender	WB:PBS^*b*^/IC^*c*^	Plasma/IC	Plasma:PBS^*d*^/IC	Serum/IC	PBMC^*e*^/IC
Case 1	HSV-2	Lesion	68	F	36.8/29.0	33.5/29.6	34.4/29.0	N/A**^*f*^**	N/A
Case 2	HSV-2	Lesion	55	M	38.4/29.4	36.9/30.1	37.5/29.5	35.3/29.0	N/A
Case 3	HSV-1	Lesion	26	F	35.8/29.1	34.3/30.8	34.6/29.5	33.6/28.7	ND/28.6
Case 4	HSV-1	Lesion	82	F	31.9/29.9	33.5/32.5	30.7/29.8	N/A	38.2/28.6
Case 5	HSV-1	Lesion	42	F	ND**^*g*^**/30.2	INV/INV**^*h*^**	39.5/31.0	38.6/29.2	ND/29.7
Case 6	HSV-2	Lesion	93	F	36.4/29.0	37.1/34.2	33.8/29.9	33.8/30.0	ND/29.0
Case 7	HSV-2	Lesion	36	M	39.2/29.4	ND/30.5	39.9/29.7	N/A	ND/29.0
Case 8	HSV-1	Lesion	45	F	39.4/30.1	INV/INV	ND/30.5	38.6/29.2	ND/30.3
Case 9	HSV-1	CSF	58	F	36.7/30.1	34.6/31.3	35.1/29.9	33.5/29.6	37.2/29.5
Case 10	HSV-1	Lesion	72	M	34.6/28.0	36.3/28.8	36.8/28.5	30.4/28.2	37.4/28.4
Case 11	HSV-2	Whole blood	62	M	33.5/30.2	32.7/31.1	32.3/29.7	31.3/28.6	ND/30.1
Case 12	HSV-1	Whole blood	28	M	36.2/29.0	35.1/29.7	35.6/29.7	N/A	ND/29.1
Case 13	HSV-1	Whole blood	83	M	38.1/29.1	35.6/29.0	35.2/28.8	35.3/29.3	ND/29.0
Case 14	HSV-1	Whole blood	37	F	34.1/29.2	31.6/30.2	32.2/29.7	31.8/29.3	ND/29.2
Case 15	HSV-1	Whole blood	52	F	39.5/30.0	ND/30.0	37.6/29.6	35.8/29.1	ND/29.7
Case 16	HSV-1	Whole blood	42	F	33.9/30.9	33.0/32	33.5/30.6	32.1/30.2	ND/30.8
Case 17	HSV-1	Whole blood	23	M	34.2/30.4	INV/INV	32.5/31.2	32.2/29.6	ND/30.7
Case 18	HSV-2	Whole blood	31	F	37/29.8	INV/INV	35.9/29.9	34.4/28.7	ND/28.7
			% Detection		94.4% (17/18)	66.7% (12/18)	94.4% (17/18)	100% (14/14)	18.8% (3/16)

^*a*^ The initial HSV-positive specimens were tested using Lyra Direct HSV 1 + 2/VZV, Quidel for Lesion samples, the DiaSorin Simplexa HSV-1/2 direct for CSF, and modified Simplexa assay Ct for whole blood (16).

^*b*^ WB/PBS: whole blood specimens were prepared in a 1:1 dilution with PBS without magnesium and calcium prior to testing.

^*c*^ IC: Simplexa internal control.

^*d*^ Plasma specimens were prepared in a 1:1 dilution with PBS without magnesium and calcium prior to testing.

^*e*^ PBMC, peripheral blood mononuclear cells.

^*f*^ Not applicable; specimen not available or insufficient volume for testing.

^*g*^ Not detected.

^*h*^ Invalid result.

**Fig 1:**
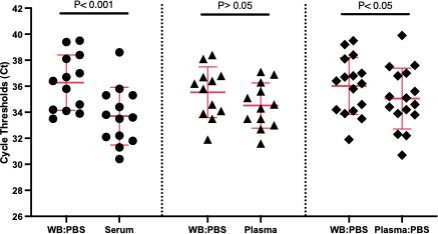
Results for the detection of HSV-1 and 2 in paired whole blood, plasma, and serum from HSV-positive individual cases. Comparison of HSV detection in paired sample types based on the distribution of overall Ct values of both targets (HSV-1 and HSV-2). The solid red line indicates the average Ct obtained for HSV-1 and HSV-2 patients in each blood compartment tested ± the standard deviation. ● For sample type WB:PBS without magnesium and calcium (PBS) vs Serum (*n* = 13, *P* < 0.001); ▲ for sample type WB:PBS vs Plasma (*n* = 12, *P* > 0.05); ♦ for sample type WB:PBS vs Plasma:PBS (*n* = 16, *P* < 0.05).

The distribution of Ct values in paired sample types from patients with HSV infections was also compared. The overall Ct values for both targets (HSV-1 and HSV-2) in WB were statistically significantly higher than those from serum (*P* < 0.001) based on the mean and between WB and the diluted plasma (*P* < 0.05) (Fig. 1). No statistical difference was observed between WB and undiluted plasma (*P* > 0.05) (Fig. 1).

### Limit of detection results for serum

The LOD was defined as the minimum concentration with detection values of ≥95% by percent positive rate and ≥95% by Probit analysis. The means ± SD Ct values and copies per reaction were also included. The LOD established by percent positive rate was 200 copies/mL for both HSV-1 and HSV-2 in serum when using the MacIntyre and MS strains, respectively. The LOD results were further subjected to Probit analysis. The 95% detection limit values were 132  ±  23 copies/mL with upper and lower 95% CIs (±95% CI) for the HSV-1 MacIntyre strain and 146  ±  17 copies/mL (±95% CI) for the HSV-2 MS strain (Table 2).

**TABLE 2:** Summary of the limit of detection results in human serum matrix using the modified Simplexa assay

Strains	Positive rate %				Probit (±95% CI)^*a*^^*b*^	
	Copies/mL	Copies/Rx	% Detection	Mean Ct ± SD (% CV)	Copies/mL	Copies/Rx
HSV-1 (MacIntyre)	200	10	100% (10/10)	36.7 ± 0.5 (2.7%)	132 ± 23	7
HSV-2 (MS)	200	10	100% (10/10)	37.1 ± 1.1 (3.0%)	146 ± 17	7

^*a*^ CI, confidence interval.

^*b*^ ±, upper/lower 95%.

### Inter- and intra-assay reproducibility for serum

The inter-and intra-assay reproducibility study was performed using a whole virus control of HSV-1 and HSV-2 diluted in pooled human negative serum at virus concentrations between 2 × 10^4^ copies/mL and 2 × 10^2^ copies/mL for 3 to 5 days (*n* = 10 to 12 replicates). The means ± SD Ct values and CV are shown in Table 3. The CV for the inter-assay assessment ranged from 1.0% to 1.4% and 1.6% to 3.0% for HSV-1 and HSV-2, respectively. There was also a high degree of intra-assay precision (Table 3). The intra-assay assessment ranged from 0.3% to 1.4% and 0.6% to 2.2% for HSV-1 and HSV-2.

**TABLE 3:** Summary of inter- and intra-assay reproducibility using the modified Simplexa assay

Analyte	Concentration (copies/mL)	Mean Ct ± SD (% CV)^*a*^	
		Inter-assay reproducibility	Intra-assay reproducibility
HSV-1	20,000	30.5 ± 0.3 (1.0)	30.3 ± 0.1 (0.3)
	400	35.5 ± 0.4 (1.1)	35.4 ± 0.5 (1.4)
	200	36.7 ± 0.5 (1.4)	36.6 ± 0.3 (0.8)
HSV-2	20,000	30.5 ± 0.5 (1.6)	30.8 ± 0.2 (0.6)
	400	36.0 ± 0.9 (2.5)	36.0 ± 0.7 (1.9)
	200	37.1 ± 1.1 (3.0)	37.1 ± 0.8 (2.2)

^*a*^ Simplexa HSV cycle threshold (Ct), standard deviation (SD), and percent coefficient of variation (% CV).

### Clinical performance comparison in serum specimens

A total of 247 serum specimens from patients with known HSV-positive lesions were tested to assess the clinical performance of the modified Simplexa assay, with a total percent agreement of 95% (95% CI, 0.92 to 0.97) between the modified Simplexa assay and the LDTs, with a κ statistic of 0.75 (95% CI, 0.62 to 0.86), demonstrating moderate agreement between assays for both targets. Overall, a total of 26 positive specimens were detected for HSV-1 (*N* = 15) and HSV-2 (*N* = 11) on the modified Simplexa assay which was also confirmed by the CRM (Table 4). A negative percent agreement of 94.4% (95% CI, 0.91 to 0.97) was observed for the HSV-1 target, while HSV-2 showed a negative percent agreement of 99.1% (95% CI, 0.97 to 1.0). Both targets had a positive percent agreement of 100% when tested using the modified Simplexa assay (Table 4). An overall discordance rate of 5.20% was found for HSV-1 and 0.81% for HSV-2 between the modified Simplexa assay and CRM using serum (Table S1). There were 13 specimens positive for HSV-1 and two positives for HSV-2 using the modified Simplexa assay that were negative by CRM.

**TABLE 4:** Clinical performance comparison in human serum specimens (*n* = 247)

Modified Simplexa assay	CRM^*a*^		(±95% CI)*^bc^*			
	Positive	Negative	PPA^*d*^	NPA^*e*^	Kappa^*f*^	Discordance rate
HSV-1						
Positive	15	13^*g*^	100%	94.4%	0.67	5.20%
Negative	0	219	(0.78–1.0)	(0.91–0.97)	(0.51–0.84)	
HSV-2						
Positive	11	2^*h*^	100%	99.1%	0.91	0.81%
Negative	0	234	(0.71–1.0)	(0.97–1.0)	(0.79–1.0)	

^*a*^ CRM was based on the combined results from the LDT 1 and LDT 2.

^*b*^ ±, upper/lower 95%.

^*c*^ CI, confidence interval.

^*d*^ PPA, positive percent agreement.

^*e*^ NPA, negative percent agreement.

^*f*^ Almost-perfect (>0.90), strong (0.80 to 0.90), moderate (0.60 to 0.79), weak (0.40 to 0.59), minimal (0.21 to 0.39), or none (0 to 0.20).

^*g*^ Thirteen samples with HSV-1 DNA detection had Ct between 36.1 to 39.4, the Ct values were from the Simplex HSV-1 and 2 Direct.

^*h*^ Two samples with HSV-2 DNA detection had Ct of 39.7 and 38.9, the Ct values were from the Simplex HSV-1 and 2 Direct.

## DISCUSSION

In the current study, we evaluated Simplexa HSV-1 & 2 Direct assay, an FDA-cleared assay that enables HSV-1 and HSV-2 differentiation using only 50 µL of CSF or a cutaneous and mucocutaneous swab sample in a sample-to-answer format, for use with additional types of blood specimens to facilitate the rapid diagnosis of disseminated HSV. We had previously evaluated this assay for use in WB specimens (16) and showed equivalent detection performance to that observed for CSF, an FDA-cleared specimen with the Simplexa assay. Our goal was to explore other specimen types to facilitate ease of collection, specimen availability, and convenience of testing without the need for sending out testing to a reference laboratory, which substantially delays results. To our knowledge, this is the first study to investigate multiple blood compartments for the detection of HSV, with a focus on serum as a specimen type. When comparing side-by-side with WB, plasma, serum, and PBMCs, the findings demonstrated that the detection of HSV-1 and HSV-2 in serum was equivalent to WB, demonstrating 100% positive percent agreement and a lower Ct value (2.6 Ct bias, *P* < 0.001). In addition to serum samples exhibiting a comparable detection performance to WB, amplification inhibition was also not observed, and no additional sample preparation steps (such as a dilution step) were required. In contrast, undiluted plasma, plasma in PBS (1:1 dilution), and PBMCs showed reduced detection of 66.7%, 94.4%, and 18.8%, respectively.

The decreased Ct value observed in serum versus WB is likely partially due to the 1:1 dilution performed for the WB specimens before testing in order to mitigate assay inhibition. Of note, serum has the additional benefits of not requiring dilution or any pre-analytical steps prior to using the sample in the modified Simplexa assay as well as being a more commonly collected specimen type. After establishing that serum was the optimal specimen type in the blood compartments tested, we carried out both a LOD analysis and a clinical comparison and determined that the assay performance in serum was comparable to that of WB, which we had previously established (16).

This study used previous HSV-1 and/or HSV-2 lesion positivity as eligibility criteria for enrollment of residual specimens, hypothesizing that specimens from these patients should have a higher positivity rate for detection of the virus than that found in random serum specimens. Interestingly, this was the finding in this study, which showed an overall HSV positivity rate of 10.5% (6.1% for HSV-1 and 4.4% for HSV-2). This rate is much higher than the expected HSV positivity rate in the general population, which has been shown to be under 1% for molecular detection when randomly enrolled for various specimen sources (35, 36). Due to this selection criteria, we can also deduce that disseminated HSV may be more common in patients presenting with lesions than previously considered since most patients in this cohort did not have HSV testing ordered for a blood specimen.

In the subset of serum samples from previously positive lesion patients, 13 specimens for HSV-1 and two specimens for HSV-2 were positive on the modified Simplexa assay but were identified as negative by the CRM. The CRM results were based on LDT 1 and LDT 2, which have higher established LODs than the modified Simplexa assay in this study. As shown in Table S1, these 15 specimens likely had low concentrations of target DNA as exhibited by the high Ct values. These results from the modified Simplexa assay were likely true positives because the samples were from patients with known HSV-positive HSV lesions at the time of specimen collection. In addition, the LOD of the LDT 2 was 250 copies/mL for HSV-1 and 500 copies/mL for HSV-2, while the modified Simplexa assay had a LOD of 132  ±  23 copies/mL for the HSV-1 MacIntyre strain and 146  ±  17 copies/mL for the HSV-2 MS strain. Considering this LOD difference, the 13 specimens that were positive for HSV-1 on the modified Simplexa assay had Ct values between 36.1 and 39.4, and the two specimens that were positive for HSV-2 had Ct values of 38.9 and 39.7, indicating these were weak positives near or below the LOD of both assays. This is supported by the results in Table 2 and Table S1, showing that the modified Simplexa assay’s direct average Ct at LOD was at a Ct of 36.7 for HSV-1 and 37.1 for HSV-2. The precision and reproducibility of the modified Simplexa assay on serum were also excellent, with both HSV-1 and 2 being reliably and reproducibly detected.

The limitation of the study is that it was only performed in a single health system. While this is the case, the core laboratory was the laboratory for multiple hospitals and also served a diverse patient population in the NYC metropolitan area.

In conclusion, the modified Simplexa assay exhibits acceptable performance for the rapid detection of HSV-1 and HSV-2 in serum, a sensitive blood specimen type that can be used for the assessment of HSV bloodstream infections. The LIAISON MDX platform’s ease of use and rapid assay turnaround time stand out as advantages. Currently, no FDA-cleared sample-to-answer assays exist on the market for blood as a specimen type, but there is a clinical need to rapidly test for HSV in blood to initiate appropriate treatment. The lack of options on the market limits testing implementation in many laboratories due to a lack of support resources required for the development of LDTs, and many laboratories need to rely on sending out testing instead. Since the modified Simplexa assay provides rapid results with a sample-to-answer workflow, it can be utilized in laboratory environments that do not necessarily have a dedicated molecular diagnostics laboratory, making this assay and the LIAISON MDX platform an appealing option to perform HSV detection in blood to more rapidly diagnose HSV bloodstream infections.

## SUPPLEMENTAL MATERIAL

Table S1 jcm.00131-24-s0001.docx Details for discordant sample analysis.

ASM does not own the copyrights to Supplemental Material that may be linked to, or accessed through, an article. The authors have granted ASM a non-exclusive, world-wide license to publish the Supplemental Material files. Please contact the corresponding author directly for reuse.

## References

[1] Gupta R, Warren T, Wald A. 2007. Genital herpes. Lancet 370:2127–2137. doi:10.1016/S0140-6736(07)61908-4 18156035

[2] Waggoner-Fountain LA, Grossman LB. 2004. Herpes simplex virus. Pediatr Rev 25:86–93. doi:10.1542/pir.25-3-86 14993516

[3] Ryder N, Jin F, McNulty AM, Grulich AE, Donovan B. 2009. Increasing role of herpes simplex virus type 1 in first-episode anogenital herpes in heterosexual women and younger men who have sex with men, 1992–2006. Sex Transm Infect 85:416–419. doi:10.1136/sti.2008.033902 19273479

[4] Looker KJ, Elmes JAR, Gottlieb SL, Schiffer JT, Vickerman P, Turner KME, Boily MC. 2017. Effect of HSV-2 infection on subsequent HIV acquisition: an updated systematic review and meta-analysis. Lancet Infect Dis 17:1303–1316. doi:10.1016/S1473-3099(17)30405-X 28843576 PMC5700807

[5] Corey L, Wald A. 2009. Maternal and neonatal herpes simplex virus infections. N Engl J Med 361:1376–1385. doi:10.1056/NEJMra0807633 19797284 PMC2780322

[6] Malm G, Forsgren M. 1999. Neonatal herpes simplex virus infections: HSV DNA in cerebrospinal fluid and serum. Arch Dis Child Fetal Neonatal Ed 81:F24–29. doi:10.1136/fn.81.1.f24 10375358 PMC1720963

[7] Harris JB, Holmes AP. 2017. Neonatal herpes simplex viral infection and acyclovir: an update. J Pediatr Pharmacol Ther 22:88–93. doi:10.5863/1551-6776-22.2.88 28469532 PMC5410863

[8] Kimberlin DW, Lin CY, Jacobs RF, Powell DA, Corey L, Gruber WC, Rathore M, Bradley JS, Diaz PS, Kumar M, Arvin AM, Gutierrez K, Shelton M, Weiner LB, Sleasman JW, de Sierra TM, Weller S, Soong SJ, Kiell J, Lakeman FD, Whitley RJ, National Institute of Allergy and Infectious Diseases Collaborative Antiviral Study Group. 2001. Safety and efficacy of high-dose intravenous acyclovir in the management of neonatal herpes simplex virus infections. Pediatrics 108:230–238. doi:10.1542/peds.108.2.230 11483782

[9] Long SS, Pool TE, Vodzak J, Daskalaki I, Gould JM. 2011. Herpes simplex virus infection in young infants during 2 decades of empiric acyclovir therapy. Pediatr Infect Dis J 30:556–561. doi:10.1097/INF.0b013e31820e3398 21304419

[10] Brady RC, Bernstein DI. 2004. Treatment of herpes simplex virus infections. Antiviral Res 61:73–81. doi:10.1016/j.antiviral.2003.09.006 14670580

[11] Lee DH, Zuckerman RA, AST Infectious Diseases Community of Practice. 2019. Herpes simplex virus infections in solid organ transplantation: guidelines from the American society of transplantation infectious diseases community of practice. Clin Transplant 33:e13526. doi:10.1111/ctr.13526 30859647

[12] Arana C, Cofan F, Ruiz P, Hermida E, Fernández J, Colmenero J, Forns X, Escude L, Cucchiari D, Moreno A, Bodro M, Herrera S, Rodriguez C, Paredes D, Diekmann F. 2022. Primary herpes simplex virus type 1 infection with acute liver failure in solid organ transplantation: report of three cases and review. IDCases 28:e01485. doi:10.1016/j.idcr.2022.e01485 35392601 PMC8980616

[13] Côté-Daigneault J, Carrier FM, Toledano K, Wartelle-Bladu C, Willems B. 2014. Herpes simplex hepatitis after liver transplantation: case report and literature review. Transpl Infect Dis 16:130–134. doi:10.1111/tid.12178 24383552

[14] Nebbia G, Mattes FM, Ramaswamy M, Quaglia A, Verghese G, Griffiths PD, Burroughs A, Geretti AM. 2006. Primary herpes simplex virus type-2 infection as a cause of liver failure after liver transplantation. Transpl Infect Dis 8:229–232. doi:10.1111/j.1399-3062.2006.00144.x 17116138

[15] Samies NL, James SH, Kimberlin DW. 2021. Neonatal herpes simplex virus disease: updates and continued challenges. Clin Perinatol 48:263–274. doi:10.1016/j.clp.2021.03.003 34030813

[16] Zhen W, Berry GJ. 2021. Herpes simplex virus-1 and -2 rapid detection in whole blood. Mol Diagn Ther 25:71–75. doi:10.1007/s40291-020-00503-5 33385297

[17] Owusu-Edusei K, Chesson HW, Gift TL, Tao G, Mahajan R, Ocfemia MCB, Kent CK. 2013. The estimated direct medical cost of selected sexually transmitted infections in the United States, 2008. Sex Transm Dis 40:197–201. doi:10.1097/OLQ.0b013e318285c6d2 23403600

[18] Lee SWH, Gottlieb SL, Chaiyakunapruk N. 2022. Healthcare resource utilisation pattern and costs associated with herpes simplex virus diagnosis and management: a systematic review. BMJ Open 12:e049618. doi:10.1136/bmjopen-2021-049618 PMC8728455 34983754

[19] Muller WJ, Zheng XT. 2019. Laboratory diagnosis of neonatal herpes simplex virus infections. J Clin Microbiol 57:e01460-18. doi:10.1128/JCM.01460-18 30602444 PMC6498033

[20] Wald A, Ashley-Morrow R. 2002. Serological testing for herpes simplex virus (HSV)-1 and HSV-2 infection. Clin Infect Dis 35:S173–S182. doi:10.1086/342104 12353203

[21] Arshad Z, Alturkistani A, Brindley D, Lam C, Foley K, Meinert E. 2019. Tools for the diagnosis of herpes simplex virus 1/2: systematic review of studies published between 2012 and 2018. JMIR Public Health Surveill 5:e14216. doi:10.2196/14216 31124465 PMC6552407

[22] Kimberlin DW, Barnett ED, Lynfield R, Sawer MH. 2021. Herpes simplex, p 407–417. In Redbook: 2021-2024. Report of the committee on infectious diseases, 32nd ed. American Academy of Pediatrics, Itasca, IL.

[23] Youssef R, Shaker O, Sobeih S, Mashaly H, Mostafa WZ. 2002. Detection of herpes simplex virus DNA in serum and oral secretions during acute recurrent herpes labialis. J Dermatol 29:404–410. doi:10.1111/j.1346-8138.2002.tb00294.x 12184636

[24] Stanberry LR, Floyd-Reising SA, Connelly BL, Alter SJ, Gilchrist MJ, Rubio C, Myers MG. 1994. Herpes simplex viremia: report of eight pediatric cases and review of the literature. Clin Infect Dis 18:401–407. doi:10.1093/clinids/18.3.401 8011823

[25] Barbi M, Binda S, Primache V, Tettamanti A, Negri C, Brambilla C. 1998. Use of Guthrie cards for the early diagnosis of neonatal herpes simplex virus disease. Pediatr Infect Dis J 17:251–252. doi:10.1097/00006454-199803000-00017 9535257

[26] Lewensohn-Fuchs I, Osterwall P, Forsgren M, Malm G. 2003. Detection of herpes simplex virus DNA in dried blood spots making a retrospective diagnosis possible. J Clin Virol 26:39–48. doi:10.1016/s1386-6532(02)00019-7 12589833

[27] Cantey JB, Mejías A, Wallihan R, Doern C, Brock E, Salamon D, Marcon M, Sánchez PJ. 2012. Use of blood polymerase chain reaction testing for diagnosis of herpes simplex virus infection. J Pediatr 161:357–361. doi:10.1016/j.jpeds.2012.04.009 22608699

[28] Levitsky J, Duddempudi AT, Lakeman FD, Whitley RJ, Luby JP, Lee WM, Fontana RJ, Blei AT, Ison MG, US Acute Liver Failure Study Group. 2008. Detection and diagnosis of herpes simplex virus infection in adults with acute liver failure. Liver Transpl 14:1498–1504. doi:10.1002/lt.21567 18825709 PMC3618973

[29] Scoble JA, Underwood MA. 2013. Whole blood polymerase chain reaction in a neonate with disseminated herpes simplex virus infection and liver failure. AJP Rep 3:67–70. doi:10.1055/s-0033-1338167 24147237 PMC3799710

[30] Simplexa HSV-1 and 2 Direct kit, [cerebrospinal fluid, cutaneous and mucocutaneous swab samples]. DiaSorin Molecular LLC. Available from: https://molecular.diasorin.com/us/kit/simplexa-hsv-1-2-direct-kit. Retrieved 18 Aug 2023.

[31] Buelow DR, Bankowski MJ, Fofana D, Gu Z, Pounds S, Hayden RT. 2013. Comparison of two multiplexed PCR assays for the detection of HSV-1, HSV-2, and VZV with extracted and unextracted cutaneous and mucosal specimens. J Clin Virol 58:84–88. doi:10.1016/j.jcv.2013.05.008 23751960

[32] Controls. Available from: https://us.diasorin.com/en/molecular-diagnostics/kits-reagents/by-format/molecular-controls. Retrieved 20 May 2024.

[33] Finney DJ, Stevens WL. 1948. A table for the calculation of working probits and weights in probit analysis. Biometrika 35:191–201. 18867423

[34] Kuypers J, Boughton G, Chung J, Hussey L, Huang ML, Cook L, Jerome KR. 2015. Comparison of the Simplexa HSV1 & 2 Direct kit and laboratory-developed real-time PCR assays for herpes simplex virus detection. J Clin Virol 62:103–105. doi:10.1016/j.jcv.2014.11.003 25464965

[35] Oh EJ, Yuk YS, Kim JK. 2021. Laboratory investigations of herpes simplex virus-1 and -2 clinical samples in Korea. Osong Public Health Res Perspect 12:385–389. doi:10.24171/j.phrp.2021.0146 34965687 PMC8721267

[36] Juhl D, Mosel C, Nawroth F, Funke AM, Dadgar SM, Hagenström H, Kirchner H, Hennig H. 2010. Detection of herpes simplex virus DNA in plasma of patients with primary but not with recurrent infection: implications for transfusion medicine? Transfus Med 20:38–47. doi:10.1111/j.1365-3148.2009.00951.x 19708895

