# Supplementary material for: Rapid diagnosis of herpes simplex virus 1 and 2 bloodstream infections utilizing a sample-to-answer platform
Source: J Clin Microbiol. 2024 Aug 12;62(9):e00131-24. doi: 10.1128/jcm.00131-24 (PMC11389144; doi:10.1128/jcm.00131-24)
Supplement: Table S1 — Details for discordant sample analysis. [file jcm.00131-24-s0001.docx]

Table 1: Details for discordant samples analysis

| Sample_ID | Simplexa HSV-1 & 2 direct / Ct | CRM | |
| --- | --- | --- | --- |
|  |  | LDT 1 | LDT 2 / Ct |
| **Serum-004** | **HSV-1 / 38.3** | **Not detected** | **Not detected** |
| Serum-011 | HSV-2 / 34.1 | HSV-2 | N/A^a^ |
| Serum-016 | HSV-1 / 36.4 | Not detected | HSV-1 / 36.2 |
| Serum-020 | HSV-1 / 35.9 | HSV-1 | N/A |
| **Serum-024** | **HSV-1 / 38.0** | **Not detected** | **Not detected** |
| Serum-035 | HSV-1 / 38.9 | Not detected | HSV-1 / 37.9 |
| **Serum-038** | **HSV-2 / 38.9** | **Not detected** | **Not detected** |
| **Serum-041** | **HSV-1 / 37.6** | **Not detected** | **Not detected** |
| Serum-044 | HSV-1 / 37.2 | HSV-1 | N/A |
| **Serum-050** | **HSV-1 / 38.2** | **Not detected** | **Not detected** |
| **Serum-054** | **HSV-1 / 39.1** | **Not detected** | **Not detected** |
| **Serum-058** | **HSV-1 / 37.1** | **Not detected** | **Not detected** |
| Serum-068 | HSV-1 / 37.0 | Not detected | HSV-1 / 36.9 |
| Serum-090 | HSV-2 / 29.2 | HSV-2 | N/A |
| **Serum-117** | **HSV-1 / 38.8** | **Not detected** | **Not detected** |
| Serum-120 | HSV-2 / 35.7 | HSV-2 | N/A |
| **Serum-124** | **HSV-1 / 37.8** | **Not detected** | **Not detected** |
| Serum-137 | HSV-1 / 38.3 | Not detected | HSV-1 / 37.7 |
| Serum-138 | HSV-1 / 33.6 | HSV-1 | N/A |
| Serum-145 | HSV-2 / 34.8 | HSV-2 | N/A |
| **Serum-148** | **HSV-1 / 39.4** | **Not detected** | **Not detected** |
| Serum-149 | HSV-1 / 37.3 | HSV-1 | N/A |
| Serum-152 | HSV-2 / 34.6 | HSV-2 | N/A |
| **Serum-153** | **HSV-2 / 39.7** | **Not detected** | **Not detected** |
| **Serum-157** | **HSV-1 / 38.1** | **Not detected** | **Not detected** |
| **Serum-163** | **HSV-1 / 37.5** | **Not detected** | **Not detected** |
| Serum-171 | HSV-2 / 34.2 | HSV-2 | N/A |
| Serum-181 | HSV-2 / 36.8 | HSV-2 | N/A |
| Serum-186 | HSV-1 / 32.6 | HSV-1 | N/A |
| Serum-192 | HSV-2 / 35.4 | Not detected | HSV-2 / 36.8 |
| Serum-207 | HSV-2 / 39.0 | Not detected | HSV-2 / 39.9 |
| Serum-209 | HSV-1 / 32.9 | Not detected | HSV-1 / 34.3 |
| **Serum-216** | **HSV-1 / 38.8** | **Not detected** | **Not detected** |
| Serum-224 | HSV-2 / 32.8 | HSV-2 | N/A |
| Serum-227 | HSV-1 / 33.7 | HSV-1 | N/A |
| Serum-229 | HSV-1 /35.1 | Not detected | HSV-1 / 36.0 |
| Serum-231 | HSV-2 / 33.1 | HSV-2 | N/A |
| Serum-234 | HSV-1 / 36.2 | Not detected | HSV-1 / 38.7 |
| Serum-237 | HSV-1 / 37.3 | Not detected | HSV-1 / 36.9 |
| **Serum-241** | **HSV-1 / 36.1** | **Not detected** | **Not detected** |
| Serum-244 | HSV-1 / 33.5 | HSV-1 | N/A |

^a^, N/A, not applicable
